# Application of citric acid can enhance the accuracy for ^13^C-urea breath tests in the diagnosis of *Helicobacter pylori* infection in Chinese patients

**DOI:** 10.1038/s41598-024-64927-3

**Published:** 2024-06-25

**Authors:** Gang Chen, Weiping Zhang, Qiaoling Wu, Qin Yu, Yongping Cai, Wenwu Luo, Jianming Xu, Lei Zhang, Rutao Hong

**Affiliations:** 1 Department of Gastroenterology, The First Affiliated Hospital of Anhui Medical University, Key Laboratory of Gastroenterology of Anhui Province, Hefei, China; 2 Department of Pathology, The First Affiliated Hospital of Anhui Medical University, Hefei, China

**Keywords:** Gastritis, Peptic ulcers

## Abstract

Previous published data have confirmed that the addition of a citric acid meal improves the accuracy of the ^13^C-urea breath test (^13^C-UBT). However, some studies have suggested that a citric acid test meal may not be necessary. Thus, the aim of this study was to evaluate the combination of a ^13^C-UBT with a citric acid meal for the diagnosis of *Helicobacter pylori (Hp)* infection in a Chinese population, particularly for patients with results in the gray zone. In this paired self-controlled study, all subjects had previously undergone ^13^C-UBTs without citric acid meals and were randomly divided into two groups based on different doses of citric acid (a low-dose citric acid group and a high-dose citric acid group, comprising meals with 0.68 g and 3.84 g citric acid powder, respectively). Positive rapid urease test (CLO) test and histology results were considered the 'gold standard'. The mean delta over baseline (DOB) value, sensitivity, specificity, positive predictive value (PPV), negative predictive value (NPV) and accuracy were compared between the two groups, particularly for patients with results in the gray zone. In total, 285 patients were tested. Of these patients, 189 were included in the low-dose citric acid group, and 96 were included in the high-dose citric acid group. Among patients with a positive ^13^C-UBT result without citric acid [delta over baseline (DOB) value ≥ 4‰, n = 174] and a negative ^13^C-UBT result without citric acid (DOB value < 4‰, n = 111), 8.0% (14/174) were false positive, and 0.9% (1/111) was false negative as determined by gold standard. Of 14 patients with false positive, 78.6% (11/14) false positive were in the gray zone of 4–10‰. However, there were no false positive ^13^C-UBT results with citric acid in the the gray zone of 4–10‰. In the comparison of the commercial ^13^C-UBT with the ^13^C-UBT in the low-dose citric acid group, the sensitivity, specificity, PPV, NPV and accuracy at 15 min were as follows: 99.1% vs. 99.1%, 97.5% vs. 88.9%, 98.2% vs. 92.2%, 98.8% vs. 98.6% and 98.4% vs. 94.7%, respectively. In the the gray zone of 4.0–10.0‰, the comparison of the commercial ^13^C-UBT with the ^13^C-UBT in the low-dose citric acid group, the sensitivity, specificity, PPV, and accuracy at 15 min were as follows: 94.4% vs. 100.0%, 100.0% vs. 0%, 100.0% vs. 75.0% and 95.8% vs. 75.0%, respectively. No significant difference was observed between the 15-min and 30-min measurement intervals in the low- and high-dose citric acid groups, including patients with results in the gray zone. The low-dose citric acid test, with an optimal measurement interval of 15 min, was highly accurate in the diagnosis of Hp infection in the Chinese population, especially for individuals with results in the gray zone.

## Introduction

The ^13^C-urea breath test (^13^C-UBT) is a noninvasive, simple, reliable and widely available test for diagnosing an active *Helicobacter pylori* (*H*. *pylori*) infection^1–7^. However, the optimal cutoff value to define whether a ^13^C-UBT result is positive or negative remains controversial^8^. Some authors have considered a gray zone, in which ^13^C-UBT results are inconclusive, to overcome this problem^9^. The gray zone, for which an exact range has not been defined, however, in two studies both by Kwon et al.^9,10^, they defined the gray zone to be 2.5–10.0‰ based on the known cutoff value of 2.5‰. Additionally, some studies have reported that most false-positive results are in the “gray zone”^10,11^.

To improve the accuracy and sensitivity of the ^13^C-UBT test, citric acid meals have been proposed as being more favorable than other test meals, such as standard semiliquid meals, semifatty acid meals and orange or apple juice^12,13^. Citric acid helps lower intragastric pH, which tends to inhibit non-*H*. *pylori* ureases, delay gastric emptying, maximize gastric distribution of the substrate and increase the contact time with *H. pylori* urease^12,14,15^. Thus, theoretically, citric acid can increase the diagnostic accuracy of the ^13^C-UBT. Several studies have investigated the efficacy of the combination of a citric acid meal with the ^13^C-UBT by comparing the ^13^C-UBT with or without citric acid, but the results were mixed^9,12,13,16–21^. Most of the published studies have focused the Western population. The study of Graham et al.^12^, one of the representative studies, revealed a dose–response relationship between urease activity and the amount of citric acid from 1 to 4 g; compared with commercial pudding, meals with 1, 2, or 4 g citric acid led to significant increases in breath ^13^CO2 activity (*p* < 0.05). Standard protocols in the United States and Europe recommend combining a citric acid meal with the ^13^C-UBT^13^. Moreover, the Maastricht VI/Florence consensus report published in 2022 provides a strong agreement and high-quality statement that citric acid is an essential component of the ^13^C-UBT^22^. However, relatively few studies assessing the combination of a citric acid meal with the ^13^C-UBT have been conducted in Asian populations, and there is no unified standard for ^13^C-UBT detection reagents.

Based on this background, the aim of this study was to evaluate the combination of the ^13^C-UBT with a citric acid meal for the diagnosis of *H. pylori* infection in the Chinese population and validate the hypothesis that a citric acid meal could improve the diagnostic accuracy of the ^13^C-UBT in Chinese individuals, particularly those with results in the gray zone.

## Methods

All methods were carried out in accordance with relevant regulations and guidelines.

### Patient population

A total of 297 patients were referred to the endoscopy unit of the Department of Gastroenterology, The First Affiliated Hospital of Anhui Medical University, between September 2022 and December 2022. The inclusion criteria were as follows: male and female patients aged 18–80 years. The exclusion criteria were (1) patients who had undergone gastric surgery; (2) patients who were administered antibiotics or consumed bismuth compounds within the last 4 weeks or who were administered proton pump inhibitors (PPIs) or H2 receptor antagonists within 2 weeks prior to the ^13^C-UBT and endoscopy; and (3) patients who had received prior *H. pylori* eradication therapy. Written informed consent was obtained from all patients, and the study was approved by the Ethics Committee of First Affiliated Hospital of Anhui Medical University. The study was registered with the Chinese Clinical Trial Registration Center (www.chictr.org.cn; registration numbers ChiCTR2200064083; registration date 26/09/2022).

### ^13^C-UBT procedure

The ^13^C-UBT, with or without a test meal, was performed within one week for all patients after fasting for a minimum of 2 h. The test meals comprised 0.68 g citric acid (low-dose group, n = 189) and 3.84 g citric acid (high-dose group, n = 96). All patients were randomly assigned to one of the two groups based on the order of the draw. A 75-mg ^13^C-urea capsule(Urea ^13^C capsule breath test Kit, Zhonghe Headway Bio-Sci & Tech Co., Ltd., Shenzhen, China, cutoff value: 4‰) and citric acid solution [Headway Citric acid solid drink, Shenzhen, China, 5 g (containing 3.84 g citric acid powder, maltose and sucrose polymer with artificial sweetener) and 2 g (containing 0.68 g citric acid powder, maltose and sucrose polymer with artificial sweetener)] dissolved in 50 mL of water were administered orally. Further breath samples were taken at 15 and 30 min. Breath samples for the commercial ^13^C-UBT (self-control 30 min) were taken at 30 min without citric acid. All patients maintained a seated position throughout the whole test. Collected samples were analyzed by the purpose-built isotope ratio mass spectrometer in the Key Laboratory of Gastroenterology of Anhui Province, The First Affiliated Hospital of Anhui Medical University. The results are expressed as the delta over baseline (DOB) value, and a DOB value greater than 4‰ was considered to indicate a positive result for *H. pylori* infection^12^. The cutoff value of 4‰, recommended by the manufacturer, was also used. ^13^C-UBT gray zone was defined from 4.0 to 10.0‰ in this study. The sensitivity, specificity, positive predictive value (PPV), negative predictive value (NPV) and accuracy of the ^13^C-UBT were evaluated at different measurement intervals (15 and 30 min), especially in the gray zone, and compared between the low-dose group and the high-dose group.

### Gastric biopsies

All patients had two biopsy samples taken from the corpus and antrum for histological assessment and underwent a rapid urease test (CLO test). Specimens were examined by a pathologist who specialized in gastroenterology and was blinded to all clinical information, including the ^13^C-UBT and CLO test results. The presence of *H. pylori* was assessed by modified Giemsa staining. In this study, H. *pylori* infection was defined as a positive result for both the CLO test and histology, which was used as the 'gold standard'. The absence of H. *pylori* infection was defined as negative results for both tests. Patients with equivocal results (one test with positive results and the other with negative results) were excluded from our analysis.

### Statistical analysis

In this study, we used paired t tests or paired Wilcoxon signed-rank tests, depending on whether the data were normally distributed. The sensitivity, specificity, PPV, NPV and overall accuracy were calculated. A p < 0.05 was considered to indicate statistical significance.

## Results

### The flow of the current study and effect of a citric acid meal on the diagnostic accuracy of the ^13^C-UBT

**Figure 1:**
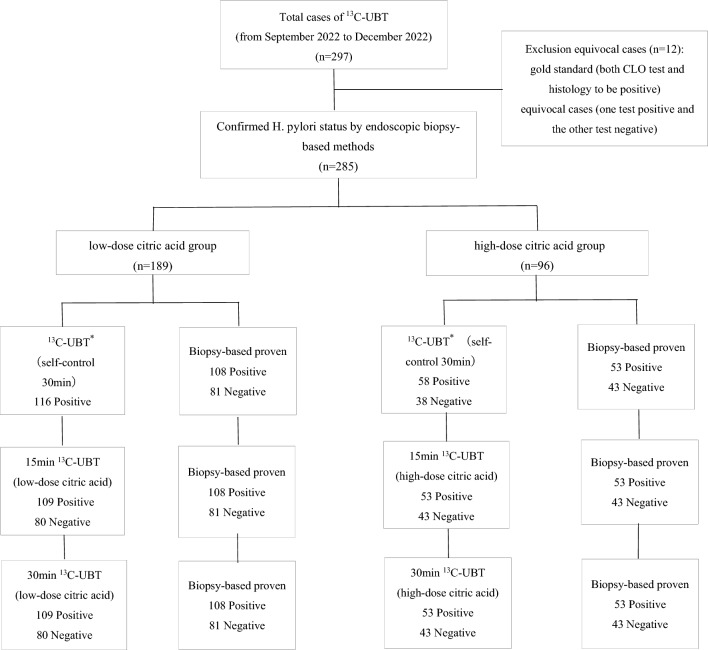
Flowchart showing the ^13^C-UBT results compared to endoscopic biopsy-based methods for confirming *H. pylori status*. ^13^C-UBT: ^13^C-urea breath test; *H. pylori*: *Helicobacter pylori*. *(Self-control 30 min): commercial ^13^C-UBT that breath samples were taken at 30 min without citric acid.

A total of 297 patients were recruited for the study. Among these patients, 12 could not be classified as having Hp infection according to our 'gold standard', i.e., patients with positive results for only one of the two tests. The remaining 285 patients were included for analysis. Of these patients, 189 were included in the low-dose citric acid group, and 96 were included in the high-dose citric acid group. The study flow is summarized in Fig. 1. Urease activity was increased in *H. pylori*-infected patients (Table 1). The mean DOB value of *H. pylori*-infected patients in the two groups, the measurement intervals were 15 min and 30 min, which were statistically (*P* < 0.0001) greater than those of the commercial ^13^C-UBT without citric acid, in which breath samples are taken at 30 min (Table 1). Although the mean DOB value of *H. pylori*-uninfected patients in the two groups at 15 min and 30 min, which were decreased than those of the commercial ^13^C-UBT without citric acid at 30 min, the decrease was not statistically (*P* > 0.05) significant (Table 1). When the diagnostic accuracy of the ^13^C-UBT was calculated, the 15-min measurement interval in the high-dose citric acid group had the highest sensitivity, specificity, PPV, NPV and accuracy for the ^13^C-UBT, with no false negative or false positive results (Table 1). When compared with the results in the 15-min measurement interval between the low- and high-dose groups, the sensitivity (99.1% vs. 100.0%), specificity (97.5% vs. 100.0%), PPV (98.2% vs. 100.0%), NPV (98.8% vs. 100.0%) and accuracy (98.4% vs. 100.0%) of the ^13^C-UBT were similar between the two groups. No significant difference was observed between the 15-min and 30-min measurement intervals in the the low- and high-dose groups (Table 1).

**Table 1:** Mean DOB, sensitivities, specificities, positive and negative predictive values, and accuracies of ^13^C-UBT with or without citric acid.

	Without citric acid (n = 189)	Low-dose citric acid (n = 189)		P value			Without citric acid (n = 96)	High-dose citric acid (n = 96)		P value		
	Self-control* (30 min)	15 min	30 min	Self-control vs. 15 min	Self-control vs. 30 min	15 min vs. 30 min	Self-control* (30 min)	15 min	30 min	Self-control vs. 15 min	Self-control vs. 30 min	15 min vs. 30 min
DOB (+) (mean ± SD)	24.00 ± 15.21	41.18 ± 27.11	36.05 ± 21.71	< 0.0001	< 0.0001	0.001	25.44 ± 16.38	49.05 ± 25.50	47.66 ± 19.95	< 0.0001	< 0.0001	0.898
DOB (−) (mean ± SD)	2.11 ± 8.69	0.79 ± 2.40	0.89 ± 2.38	0.495	0.573	0.239	1.22 ± 2.22	0.44 ± 0.36	0.90 ± 0.83	0.129	0.674	0.002
Sensitivity (%)	99.1 (107/108)	99.1 (107/108)	99.1 (107/108)	> 0.999	> 0.999	> 0.999	100.0 (53/53)	100.0 (53/53)	100.0 (53/53)	> 0.999	> 0.999	> 0.999
Specificity (%)	88.9 (72/81)	97.5 (79/81)	97.5 (79/81)	0.056	0.056	> 0.999	88.4 (38/43)	100.0 (43/43)	100.0 (43/43)	0.055	0.055	> 0.999
PPV (%)	92.2 (107/116)	98.2 (107/109)	98.2 (107/109)	0.060	0.060	> 0.999	91.4 (53/58)	100.0 (53/53)	100.0 (53/53)	0.058	0.058	> 0.999
NPV (%)	98.6 (72/73)	98.8 (79/80)	98.8 (79/80)	> 0.999	> 0.999	> 0.999	100.0 (38/38)	100.0 (43/43)	100.0 (43/43)	> 0.999	> 0.999	> 0.999
Accuracy (%)	94.7 (179/189)	98.4 (186/189)	98.4 (186/189)	0.087	0.087	> 0.999	94.8 (91/96)	100.0 (96/96)	100.0 (96/96)	0.059	0.059	> 0.999

*(Self-control 30 min): commercial ^13^C-UBT that breath samples were taken at 30 min without citric acid.

(+): *H. pylori* status gold standard positive; (−): *H. pylori* status gold standard negative.

### Diagnostic accuracy of the ^13^C-UBT for patients with results in the gray zone of 4–10‰

**Figure 2:**
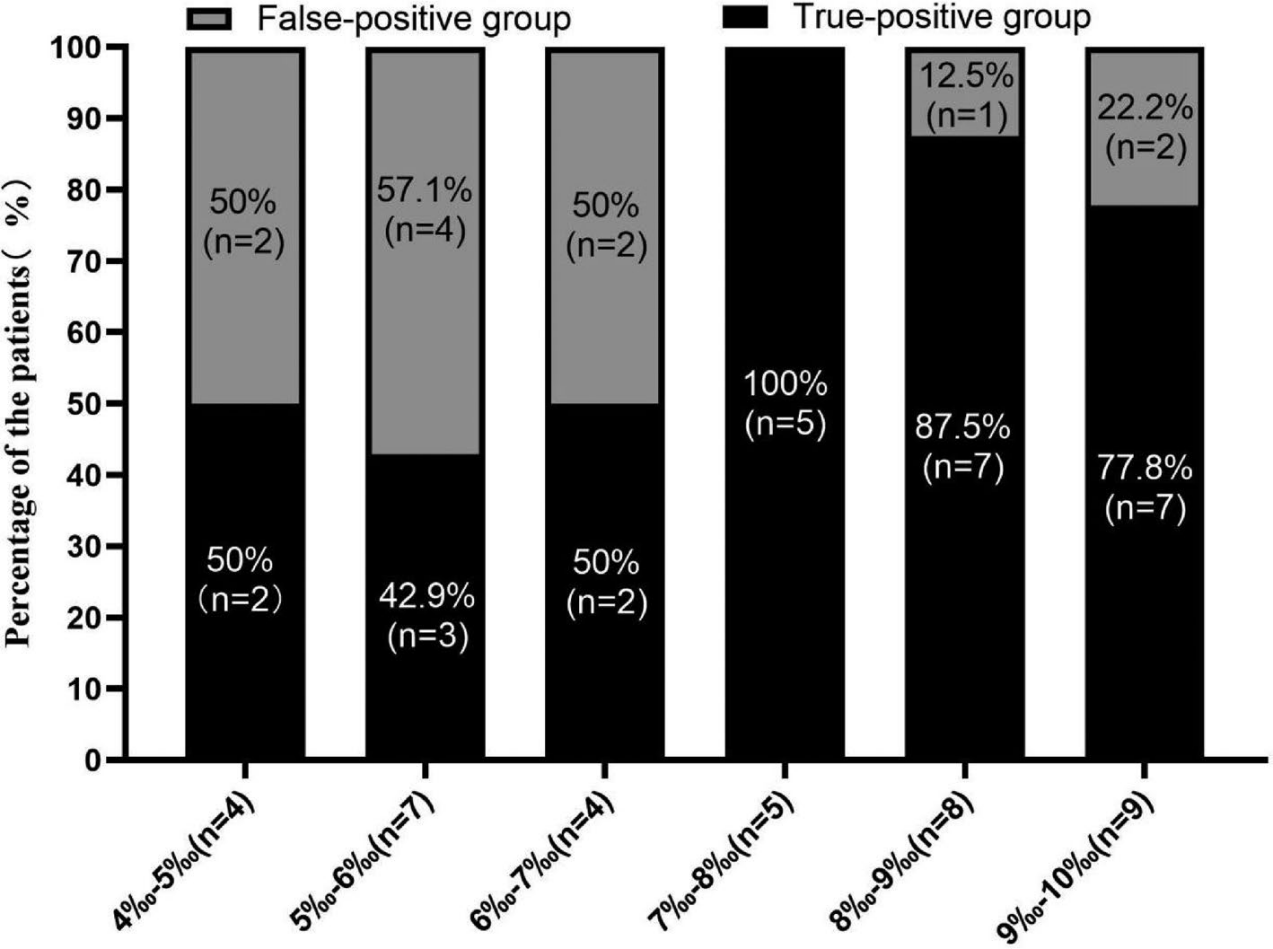
False-positive ^13^C-UBT result rates in the gray zone of 4–10‰.

Of the 285 participants, 13%(37/285) were in the the gray zone of 4–10‰, which 64.9% (24/37) were in the low-dose citric acid group and 35.1% (13/37) were in the high-dose citric acid group. Among patients with a positive ^13^C-UBT result without citric acid (≥ 4‰, n = 174) and a negative ^13^C-UBT result without citric acid (< 4‰, n = 111), 8.0% (14/174) were false positive, and 0.9% (1/111) was false negative as determined by 'gold standard'. Of 14 patients with false positive, 78.6% (11/14) false positive were in the gray zone of 4–10‰. The false-positive rates of ^13^C-UBT at cutoff values between 4 and 10‰ ranged from 12.5 to 57.1% (Fig. 2). However, there were no false positive ^13^C-UBT results in the the gray zone of 4–10‰ with citric acid, including low-dose and high-dose groups. In the the gray zone, the mean DOB value of *H. pylori*-infected patients in the two groups, the measurement intervals were 15 min and 30 min, which were statistically (*P* = 0.008) greater than those of the commercial ^13^C-UBT without citric acid, in which breath samples are taken at 30 min (Table 2). When the diagnostic accuracy of the ^13^C-UBT in the gray zone was calculated, the 15-min measurement interval in the high-dose citric acid group had the highest sensitivity, specificity, PPV, NPV and accuracy of 100.0% (8/8), 100.0% (5/5), 100.0% (8/8), 100.0% (5/5), and 100.0% (13/13), respectively (Table 2). The commercial ^13^C-UBT breath samples taken at 30 min without citric acid in the gray zone showed the lowest specificity, PPV and accuracy of 0% (0/5), 61.5% (8/13) and 61.5% (8/13), respectively (Table 2). When compared with the results in the 15-min measurement interval between the low- and high-dose groups in the gray zone, the sensitivity (94.4% vs. 100.0%), specificity (100.0% vs. 100.0%), PPV (100.0% vs. 100.0%), NPV (87.5% vs. 100.0%) and accuracy (95.8% vs. 100.0%) of the ^13^C-UBT were similar between the two groups. No significant difference was observed between the 15-min and 30-min measurement intervals in the the low- and high-dose groups (Table 2).

**Table 2:** Mean DOB, sensitivities, specificities, positive and negative predictive values, and accuracies of ^13^C-urea breath test with or without test meals in the gray zone (Value of ^13^C-UBT: 4.0–10.0‰).

	Without citric acid (n = 24)	Low-dose citric acid (n = 24)		P value			Without citric acid (n = 13)	High-dose citric acid (n = 13)		P value		
	Self-control* (30 min)	15 min	30 min	Self-control vs. 15 min	Self-control vs. 30 min	15 min vs. 30 min	Self-control* (30 min)	15 min	30 min	Self-control vs. 15 min	Self-control vs. 30 min	15 min vs. 30 min
DOB( +) (mean ± SD)	7.75 ± 1.79	24.28 ± 18.99	20.08 ± 13.90	< 0.0001	< 0.0001	0.052	6.85 ± 1.98	28.91 ± 13.90	32.91 ± 14.76	0.008	0.008	0.219
DOB(-) (mean ± SD)	NA(n = 0)	0.51 ± 0.47	1.08 ± 1.14	NA	NA	0.469	NA(n = 0)	0.38 ± 0.34	0.84 ± 0.97	NA	NA	0.375
Sensitivity (%)	100.0% (18/18)	94.4% (17/18)	100.0% (18/18)	> 0.999	> 0.999	> 0.999	100.0% (8/8)	100.0% (8/8)	100.0% (8/8)	> 0.999	> 0.999	> 0.999
Specificity (%)	0% (0/6)	100.0% (6/6)	100.0% (6/6)	0.002	0.002	> 0.999	0% (0/5)	100.0% (5/5)	100.0% (5/5)	0.008	0.008	> 0.999
PPV (%)	75.0% (18/24)	100.0% (17/17)	100.0% (18/18)	0.033	0.029	> 0.999	61.5% (8/13)	100.0% (8/8)	100.0% (8/8)	0.111	0.111	> 0.999
NPV (%)	NA(0/0)	85.7% (6/7)	100.0% (6/6)	NA	NA	> 0.999	NA(0/0)	100.0% (5/5)	100.0% (5/5)	NA	NA	> 0.999
Accuracy (%)	75.0% (18/24)	95.8% (23/24)	100.0% (24/24)	0.097	0.022	> 0.999	61.5% (8/13)	100.0% (13/13)	100.0% (13/13)	0.039	0.039	> 0.999

*(Self-control 30 min): commercial ^13^C-UBT that breath samples were taken at 30 min without citric acid.

(+): *H. pylori* status gold standard positive; (−): *H. pylori* status gold standard negative.

*NA* not applicable.

## Discussion

As noted earlier, this was a paired self-controlled study based on the 'gold standard' to determine whether a citric acid meal could improve the diagnostic accuracy of the ^13^C-UBT in Chinese individuals, particularly those with results in the gray zone. We found that the sensitivity, specificity, PPV, NPV and accuracy of the citric acid group were significantly higher than those of the self-control group, particularly for patients with results in the gray zone. However, no statistically significant difference was observed between the low- and high-dose groups. Moreover, there was no significant difference between the 15-min and 30-min measurement intervals in the citric acid group.

Furthermore, inconsistent efficacy of the combination of a citric acid meal with the ^13^C-UBT has been reported worldwide, and most of the studies were based on Western data^9,12,13,16–21^. The present paired self-controlled study reported that the mean ^13^C-UBT value, sensitivity, specificity, PPV, NPV and accuracy of the citric acid group (both low- and high-dose groups) were significantly higher than those of the self-control group. Nevertheless, there was no significant difference between the 15-min and 30-min measurement intervals in the citric acid group (both low- and high-dose groups). This result suggests that the use of a citric acid test meal could potentially increase the accuracy of the ^13^C-UBT and allow for a shorter test duration. The fact that urease activity was markedly enhanced after the ingestion of urea in citric acid suggested that citric acid could increase urea hydrolysis by *H. pylori* and lower intragastric pH, which tends to inhibit non-*H. pylori* ureases^14^. Urea might also delay gastric emptying and maximize the distribution of the substrate within the stomach, thereby enhancing the contact area and time between the bacteria and the substrate^1,8^. Graham et al.^12^ conducted a single-center, paired self-controlled study in the United States and evaluated the effect of the combination of citric acid test meals (1 g, 2 g and 4 g citric acid dissolved in 200 mL of water) with the ^13^C-UBT (125 mg of ^13^C-urea, cutoff value 2.4 DOB, n = 46) and revealed a dose‒response relationship between the mean DOB value and the amount of citric acid (increasing from 1 to 4 g) compared with commercial pudding (*p* < 0.05). In addition, a 2019 prospective study by Kwon et al.^9^, on the effect of citric acid on the accuracy of the ^13^C-UBT after *H. pylori* eradication therapy in Korea reported that the mean ^13^C-UBT value of the citric acid group was significantly (*p* < 0.001) higher than that of the control group; however, interestingly, the inclusion of a citric acid meal did not increase the diagnostic accuracy or specificity of the ^13^C-UBT after *H. pylori* eradication therapy. In 2000, Wong et al.^21^ conducted a single-center, paired self-controlled clinical study in Hong Kong, China and revealed that the ^13^C-UBT with or without a citric acid test meal produced highly accurate and reliable results.

Some studies suggested that most false-positive results are in the gray zone and should be considered inconclusive^10,11^. Moreover, the interpretation of the gray zone, for which an exact range has not been defined, remains a controversial issue. A study by Kwon et al.^10^, 1891 patients who had received *H. pylori* eradication therapy in Korea, they defined the gray zone to be 2.5–10.0‰ based on the known cutoff value of 2.5‰, and the false-positive rates of ^13^C-UBT value in the gray zone ranged from 6.7% to 77.3%. However, citric acid had no beneficial effect on the diagnostic validity of results in the gray zone. As we known, there were no reports in the Chinese population on the combination of a citric acid meal with the ^13^C-UBT in the gray zone. Therefore, our study is the first to evaluate the ^13^C-UBT for results in the gray zone for the diagnosis of *H. pylori* infection in the Chinese population. As we expected, the combination of the ^13^C-UBT with a citric acid meal showed reliable and excellent results in the diagnosis of *H*. pylori infection in the Chinese population. We found that the citric acid group had a significantly reduced false-positive rate compared with the group who underwent a commercial ^13^C-UBT and had results in the gray zone. This suggested that the combination of the ^13^C-UBT with a citric acid meal benefited the diagnostic validity, which became more prominent for patients in the gray zone. The discrepancy of results between our study and a study by Kwon et al. can be explained as follows. First, the gold standard for diagnosing *H*. *pylori* infection is different. In Kwon et al.’s study^9^, the 'gold standard' for *H*. *pylori* infection is either positive CLO test or histology results. The number of patients who underwent histology was only 10.1% (122/1207) of all enrolled patients; moreover, only 92 participants were evaluated by both histology and the CLO test, which may have affected the relatively low accuracy of the ^13^C-UBT. However, in our study, 285 patients were included for analysis and all patients were evaluated by endoscopic biopsy methods(both histology and the CLO test). Positive CLO test and histology results were considered the 'gold standard' in our study. Second, it should be noted that differences in research methods might be a potential explanation. Our approach was the same as that in Graham’s study^12^; in this paired self-control study, all subjects previously underwent the ^13^C-UBT without citric acid meals and were blindly assigned citric acid groups receiving different doses (citric acid powder dissolved in 50 mL of water). Nevertheless, Kwon et al.^9^ randomly divided patients into two groups, one group with a citric acid meal and the other without a citric acid meal (4 g in 200 mL of water containing 50 g glucose polymer with artificial sweetener). Third, the study population is different. In a study by Kwon et al.^9^, all patients were after *Helicobacter pylori* Eradication Therapy. In our study, all patients who had not received prior *H. pylori* eradication therapy. Fourth, other potential explanations include differences in the distribution, delivery and emptying of ^13^C-urea and ethnic differences in gastric emptying^23^.

This study has several limitations. First, this was a paired self-controlled study conducted at a single center with a relatively modest sample size; hence, a well-designed, prospective, multicenter, randomized controlled study is needed to verify the results. Third, the effect of citric acid meals on the accuracy of the ^13^C-UBT after *H. pylori* eradication therapy still needs to be evaluated.

Citric acid solutions are bitter and unpleasant. Our data suggested that the ability to achieve favorable results was similar between the low- and high-dose citric acid groups at the 15-min measurement interval. Low-dose citric acid may be a good alternative to improve palatability for patients. Additionally, a citric acid test meal can reduce the false positive rate and improve accuracy; to some extent, it can also reduce the unnecessary psychological burden and medical expenses for patients. To date, there are no reports that the addition of citric acid disturbs ^13^C-UBT results. Overall, citric acid meals can increase the sensitivity, specificity, and accuracy of the ^13^C-UBT in detecting *H. pylori* infection, especially for patients with results in the gray zone, and there is little justification for not using citric acid as an adjuvant to the ^13^C-UBT^24^. Taken together, our findings demonstrated that a low-dose citric acid meal, with an optimal measurement interval of 15 min, was highly accurate in the diagnosis of *H. pylori* infection in the Chinese population, especially for patients with results in the gray zone. The combination of the ^13^C-UBT with a citric acid meal may become the standard protocol in the Chinese population.

## Data Availability

The datasets generated and analyzed during the current study are available from the corresponding author on reasonable request.
